# The expression and significance of IDH1 and p53 in osteosarcoma

**DOI:** 10.1186/1756-9966-29-43

**Published:** 2010-05-07

**Authors:** Xiang Hu, Ai-Xi Yu, Bai-Wen Qi, Tao Fu, Gang Wu, Min Zhou, Jun Luo, Jun-Hua Xu

**Affiliations:** 1 Department of Orthopedics, Zhongnan Hospital of Wuhan University, No 169 Donghu Road, Wuchang District, 430071, Wuhan, China; 2 Department of pathology, Zhongnan Hospital of Wuhan University, Wuhan, China

## Abstract

**Background:**

To detect the expression of isocitrate dehydrogenase 1 (IDH1) and transformation-related protein 53 (p53) in osteosarcoma and analyze the correlation between them and the clinico-pathological features.

**Methods:**

The expressions of IDH1 and p53 were detected in human osteosarcoma cell lines (MG-63 and U2OS) by immunocytochemistry, Real-time PCR and Western Blotting. The expressions of IDH1 and p53 in formalin-fixed paraffin-embedded tissue sections from 44 osteosarcoma patients were determined by immunohistochemistry, and the correlation between them and clinicopagthological features were analyzed. None of these patients received chemotherapy prior to surgery.

**Results:**

IDH1 is detected in osteosarcoma cell lines and biopsies. IDH1 expresses higher in U2OS cells with wild type p53 than in MG-63 cells with mutation p53. IDH1 correlates with histological Rosen grade and metastasis negatively. P53 correlates with histological Rosen grade, metastasis and overall survival in clinical osteosarcoma biopsies. Osteosarcoma patients with High IDH1 expression have a very high p53 expression.

**Conclusion:**

IDH1 may correlate with p53 and be a candidate biomarker for osteosarcoma correlate with histological Rosen grade and metastasis.

## Background

Osteosarcoma (OS) is the most current primary malignant bone tumor in children and adolescents. Presently, 60% of the affected patients are cured by wide resection of the tumor and aggressive adjuvant chemotherapy [1,2]. However, around 40% of the individuals with metastases still emerge which normally exhibit resistance to cytostatics and acquire "second malignancies" [3]. The identification of biomarkers linked to clinicopagthological features and development of this disease is crucial for the diagnosis and treatment of these patients [4,5].

Genetic alterations caused either by lost of heterozygosity or by mutations have been reported in osteosarcoma. Such alterations can occur in tumor suppressor genes, such as tumor protein 53(p53) and phosphates and tensin homolog (PTEN). The p53 mutations occurs commonly in primary osteosarcoma [6]. It is implicated in the pathogenesis of various human malignancies through loss of function mutations [7,8]. P53 contributes to the development, life expectancy, and overall fitness of an organism except for its role in protecting against cancer development [9]. PTEN is known to be the most highly mutated tumor suppressor gene after p53 [10]. It plays an important role in regulating proliferation, migration, survival, cell invasion and tumor angiogenesis [11,12]. Freeman et al. [13] reported that loss of PTEN was a common occurrence in osteosarcoma. It was further demonstrated that PTEN can control p53 half-life independent via a currently unknown mechanism [14]. In addition, mutations of tumor-suppressor retinoblastoma gene (Rb) in osteosarcoma are associated with a poor prognosis [15]. However, none of these alterations can characteristically reflect the biologic nature or clinical features of all osteosarcomas.

IDH1 is a cytosolic NADP-dependent isocitrate dehydrogenase. It catalyzes decarboxylation of isocitrate into alpha-ketoglutarate [16]. Shechter et al. [17] described that the activity of IDH1 is coordinately regulated through the cholesterol and fatty acid biosynthetic pathways, suggesting that IDH1 provides the cytosolic NADPH required by these pathways. Memon et al. [18] found that expression of IDH1 was downregulated in a poorly differentiated bladder cancer cell line compared with a well-differentiated bladder cancer cell line. Tissue biopsies of late-stage bladder cancers also showed IDH1 downregulation compared with early-stage bladder cancers. Yan et al. [19] described that mutations of NADP (+)-dependent isocitrate dehydrogenases encoded by IDH1 and IDH2 occur in a majority of several types of malignant gliomas.

Interestingly, Parsons et al. [20] found that IDH1 mutations in human glioblastoma had a very high frequency of p53 mutation. Mutation of the IDH1 gene was also strongly correlated with a normal cytogenetic status [21]. IDH1 appears to function as a tumor suppressor that, when mutationally inactivated, contributes to tumorigenesis [21,22]. But, there is no study on the expression of IDH1 in osteosarcoma. As to the previous study on IDH1 and p53, we are also curious intensively about the correlation between IDH1 and p53. So, we developed a study to characterize the expression and significance of IDH1 and p53 in osteosarcoma cell lines (MG63 and U2OS) as well as in clinical patient biopsies.

## Methods

### Cell lines and cell culture

The human osteosarcoma (OS) cell lines MG63 and U2OS (obtained from ATCC through LGC Promochem, Wesel, Germany) were cultured in RPMI 1640 media (Sigma, USA) with 10% fetal bovine serum (Amresco, USA) and antibiotics. Cells were cultured according to standard techniques in cell culture flasks in a humidified incubator in 5% CO_2 _atmosphere.

### Immunocytochemistry

Cell lines were grown on coverslips treated with the appropriate growth media in 24 well cluster plates. Cells were fixed in 2% formaldehyde in 0.1 mol/L phosphate-buffered saline (PBS, pH 7.4) for 20 min at room temperature and subsequently washed three times in PBS. Coverslips were permeabilized with 0.1% Triton X-100 for 15 min and blocked in 3% H_2_O_2_-methyl alcohol for 15 min. The coverslips were incubated with anti-IDH1 rabbit polyclonal antibody (protein technology group, USA) in blocking buffer overnight at 4°C. Coverslips were then incubated with an anti-rabbit secondary antibody and peroxidase-conjugated strepavidin-biotin complex (Santa Cruz, CA, USA) at 37°C for 45 min at room temperature in the dark [23]. Immunoreactivity was visualized with diaminobenzidine (DAB) (Zymed, South San Francisco, CA). Negative controls were obtained by omitting the primary antibody. Slides were scanned using a microscopy (Carl Zeiss AG, Germany), images were recorded using a digital camera (DC 500, Leica) and the Leica FW 4000 software and images were processed using Adobe Photoshop.

### Real-time PCR

Cellular total RNA from OS cells was extracted with TRIZOL Reagent (Invitrogen, Carlsbad, CA, USA). The concentration of RNA was determined by the absorbance at 260 nm and the purity was determined by the 260/280 ratio with a BioPhotometer(Eppendorf, Hamburg, Germany). For each reaction, 1 μg RNA was reverse-transcribed with random primer by ReverTra Ace (Toyobo, Osaka, Japan). RNA quality and efficiency of reverse transcription were examined by PCRs from each 1 μl cDNA according to the manufacturer's recommendations [24]. The mRNA expression of IDH-1, p53 and internal control geneβ-actin was quantified by Real-time PCR Detection System (SLAN, HONGSHI) with SYBR Green I (Toyobo, Osaka, Japan). As PCR was performed according to standard procedures [24,25] after optimization, PCR-reactions were within the exponential range of amplification. The gene-specific exon-spanning PCR primer pair for IDH1 was 5'-TCAGTGGCGGTTCTGTGGTA-3',5'-CTTGGTGACTTGGTCGTTGGT-3', and for p53^7-8 ^was 5'-CAGCCAAGTCTGTGACTTGCACGTA C-3',5'-CTATGTCGAAAAGTGTTTCTGTCATC-3', and for β-actin was 5'-GTCCACCGCAAATGCTTCTA-3',5'-TGCTGTCACCTTCACCGTTC-3'. The sequences of the primers were checked by Nucleotide BLAST for specific gene amplification. Omission of cDNA template was used as a negative control. Triplicate measurements were made of all genes in each patient and data of mean were used. For relative quantification of genes expression level, standard curves were built by considering at least three points of a ten-fold dilution series of cDNA in water. Relative gene expression data are given as the n-fold change in transcription of the target genes normalized to the endogenous control in the same sample.

### Protein extraction and Western blot

Lysates of cells were prepared using lysis buffer from the Dual-Luciferase assay kit (Promega) according to the manufacturer's recommendations. The lysates were collected and centrifuged at 12,000 g for 10 min at 4°C. The protein in the supernatants were pooled together and stored at -80°C until concentration analyzed by the BCA Protein Assay Kit (Sangon, Shanghai, China). After being heated at 99°C for 5 min in loading buffer, equal volume of tissue lysates (40 μg of protein) were then loaded for sodium dodecyl sulphate-polyacrylamide gel electophoresis (SDS-PAGE) analysis and subsequently electrotransferred from the gels onto a polyvinylidene difluoride (PVDF) membranes (Millipore, MA, USA). The transferred membranes were blocked with 5% skim milk in Tris-buffered saline with 0.05% Tween (TBST) and washed six times in TBST. IDH1 and p53 proteins were detected by the rabbit polyclonal antibody for IDH1 (protein technology group, USA) or p53 (Santa Cruz, CA, USA). β-actin proteins were recognized by the β-actin-specific monoclonal mouse IgG (Santa Cruz, CA, USA). Antibodies were diluted according to the manufacture direction and were incubated overnight at 4°C followed by incubating with peroxidase-conjugated goat anti-rabbit immunoglobulin (Santa Cruz, CA, USA, 1:2000) in TBST for 1 h. Signals were developed using enhanced chemiluminescent reagent (Pierce Biotechnology, Rockford, IL, USA). β-actin is used as the internal loading control. The band intensity was analyzed using Quantity One software (Bio-Rad, Hercules, and CA). Relative expression was calculated as the intensity ratio of target protein to that of β-actin.

### Tissue specimens and clinical data

Fifty-one formalin-fixed, paraffin-embedded osteosarcoma biopsies (before the administration of neo-adjuvant chemotherapy) were collected according to the Chinese national ethical guidelines ('Code for Proper Secondary Use of Human Tissue', Chinese Federation of Medical Scientific Societies). Because of limitations in available tumor material and following up information, only 44 of these osteosarcoma tumor samples including 32(72.7%) males and 12(27.3%) females with mean age(M ± SD) of 25.25 ± 13.61 years (range 9-61) were amenable for use in this study. Patients were followed until death from disease, or until the latest clinical therapy at the end of this study. The mean following-up time(M ± SD) were 4.26 ± 1.99 years (range 0.5-9.0). All patients consisted with the diagnostic criteria of osteosarcoma defined in the World Health Organization classification. Written informed consent was obtained from each patient before entering into this study. Clinical information was available in Table 1.

**Table 1:** Clinical Features

Features	Total(N)	Percentage
**Age(year)**		
<12	3	6.8%
13--20	14	31.8%
21--30	8	18.2%
31--40	14	31.8%
41-	5	11.4%
**Sex**		
Male	32	72.7%
Female	12	27.3%
**Localization of primary tumor**		
Distal femur	13	29.5%
Proximal tibia	11	25.0%
Humerus	3	6.8%
Tibia diaphysis	5	11.4%
Femur diaphysis	7	15.9%
Other	5	11.4%
**Histological type**		
Osteoblastic	29	65.9%
Small cell	1	2.3%
Chondroblastic	6	13.6%
Teleangetatic	1	2.3%
Round cell	2	4.5%
Fibroblastic	4	9.1%
Mixed	1	2.3%
Histological Rosen grade*		
1	5	11.3%
2	16	36.4%
3	16	36.4%
4	7	15.9%
1+2	21	47.7%
3+4	23	52.3%
**Metastasis**		
no	23	53.3%
lung	17	38.6%
other	4	9.1%

*As described previously [30-32], Grade 1: <50% tumor necrosis; Grade2: 50% to 90% tumor necrosis; Grade 3: > 90% tumor necrosis;Grade 4: 100% tumor necrosis.1+2: low grade; 3+4: high grade.

### Immunohistochemistry for biopsies

Sections were cut from formalin-fixed, paraffin-embedded granulation tissue. They were hydrated through graded alcohols. For antigen unmasking, sections were treated in trypsin solution for 10 min at 37°C. Sections were then washed with deionized water and incubated with 3% H_2_O_2 _for 5 min. They were incubated in anti-IDH1 mAb (protein technology group, USA) or anti-p53 mAb (Santa Cruz, CA, USA) for 1 h at room temperature, followed by secondary antibody and peroxidase-conjugated strepavidin-biotin complex (Santa Cruz, CA, USA) at 37°C for 30 min. Immunoreactivity was visualized with diaminobenzidine (DAB) (Zymed, South San Francisco, CA). Negative controls were obtained by omitting the primary antibody.

### Evaluation of immunohistochemistry

The slides were evaluated under the microscope. The percentage of cells showing positive nuclear staining for p53 was calculated by reviewing the entire spot. For IDH1, cytoplasmic immunostaining was considered to be positive. The staining patterns were classified into scales on the percentage of cells with positive staining [26,27]: 0, absence of nuclear (or cytoplasmic) stained cell; 1, <10% positive cells; 2, 10-25% positive cells; 3, 26-50% positive cells; 4, 51-75% positive cells; 5, >75% positive cells. For statistical analysis, osteosarcoma patients were also grouped as either low-staining group (scale 0-3: positive staining ≤ 50%) or high-staining group (scale 4, 5: positive staining >50%). Biopsy Stained less than 10% was considered as a negative result, while stained more than 10% was considered as a positive one. At least 5 separated foci of neoplastic infiltration in each biopsy were analyzed. Assessment of Immunostaining intensity was completed by three independent observers. Slides were scanned using a microscopy (Carl Zeiss AG, Germany), images were recorded using a digital camera (DC 500, Leica) and the Leica FW 4000 software and images were processed using Adobe Photoshop.

### Statistical analysis

All statistical analyses were performed using the SPSS 13.0 software package for Windows (SPSS Inc., Chicago, IL, USA). The values for the description of the statistical significance of IDH1 or p53 expression in different osteosarcoma cell lines were calculated by independent, two-tailed Student's t-tests after the Levine's Test for Equality of Variances. Mann-Whitney U was used for unnormal continuous variables. Categorical variables were analyzed by the Pearson Chis-square test and Fisher's exact test. Associations were assessed by Pearson correlation coefficient for normal data or Spearman's correlation coefficient for nonnormal data. Kaplan-Meier test was used for analysis of survival versus IDH1 and survival versus p53 expression. *P *< 0.05 was considered as statistically significant. *P *< 0.01 was considered as statistically highly significant.

## Results

### IDH1 expresses higher in U2OS compared with in MG63

**Figure 1:**
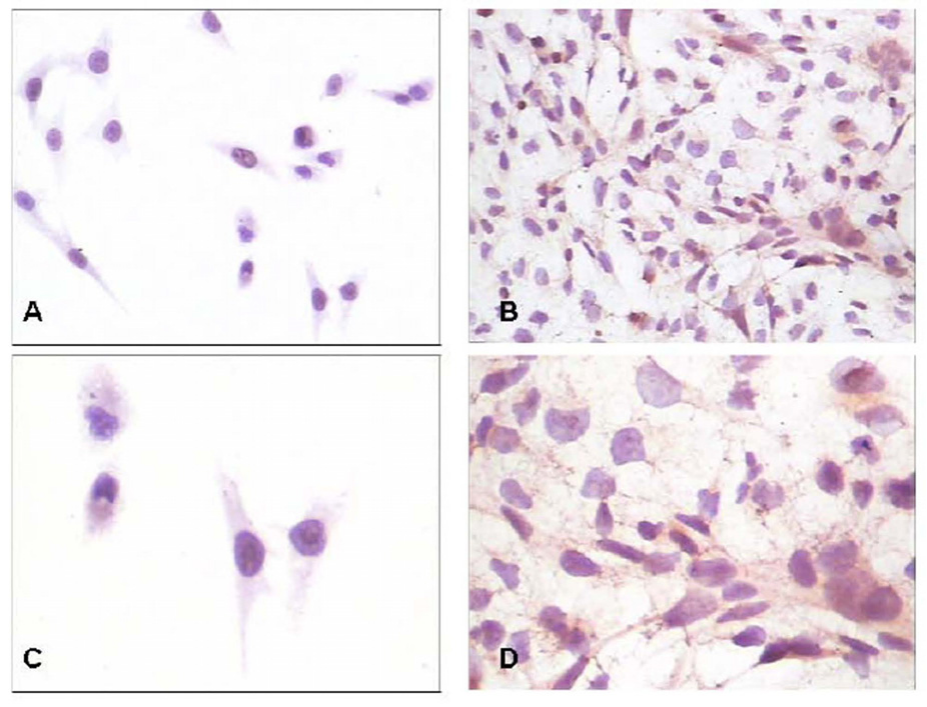
**The immunocytochemistry of IDH1 in MG63 and U2OS**. IDH1 is specifically detected in the cytoplasm of both osteosarcoma cell lines MG63 and U2OS.(A) Expression of IDH1 in U2OS, × 200; (B) Expression of IDH1 in MG63,× 200; (C) Expression of IDH1 in U2OS,× 400; (D) Expression of IDH1 in MG63,× 400.

**Figure 2:**
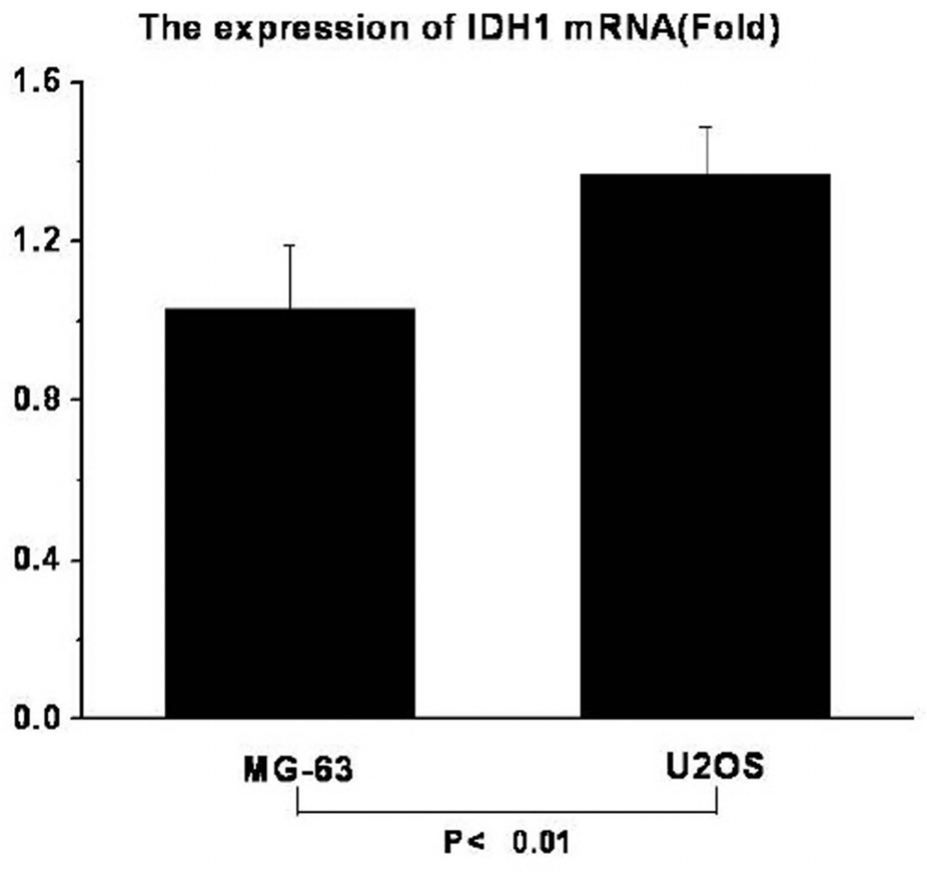
**The mRNA levels of IDH1 in MG63 and U2OS (on fold)**. The mRNA levels of IDH1 is higher in U2OS than in MG63(*P *< 0.01).

**Figure 3:**
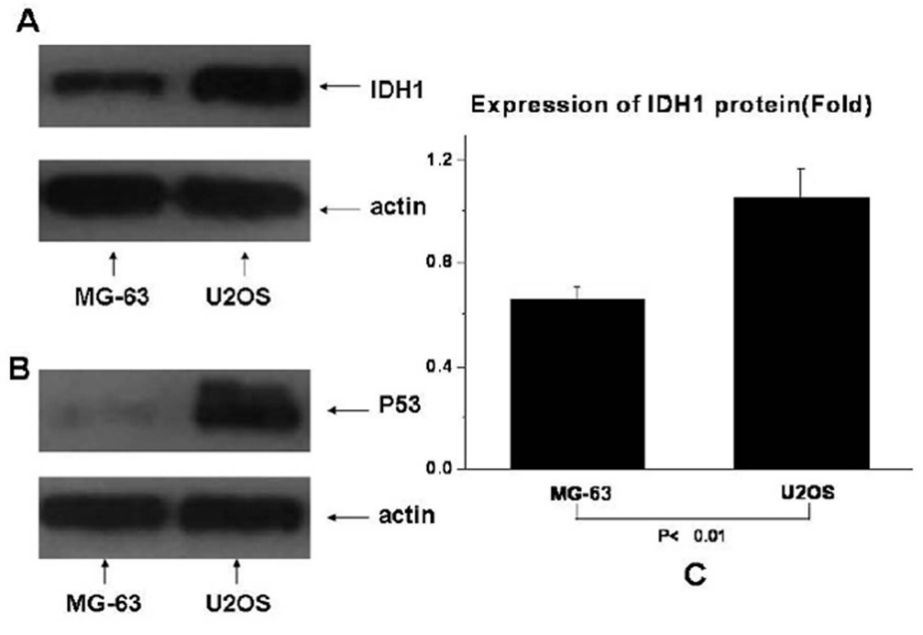
**The protein expression levels of IDH1 and p53 in U2OS and MG63**. MG63 demonstrates no detectable p53 while U2OS cells demonstrates a high expressed p53. IDH1 expresses higher in U2OS than in MG63 at the protein level(*P *< 0.01).

Expression of IDH1 is specifically detected in the cytoplasm of both osteosarcoma cell lines U2OS and MG63 (Fig. 1). The expression of IDH1 mRNA is higher in U2OS than in MG63, and *P *< 0.01(Fig. 2). The western blotting result(Fig. 3A, Fig. 3C) shows that IDH1 is highly expressed in U2OS(*P *< 0.01), and these results corroborate the immunocytochemistry(Fig. 1).

### Expression of p53 in U2OS and MG63

Consistent with data published previously [28,29]; our MG63 demonstrates no detectable p53 while U2OS demonstrates high expressed p53. The result is shown in Fig. 3B.

### IDH1 correlates with histological Rosen grade and metastasis in clinical osteosarcoma biopsies

**Figure 4:**
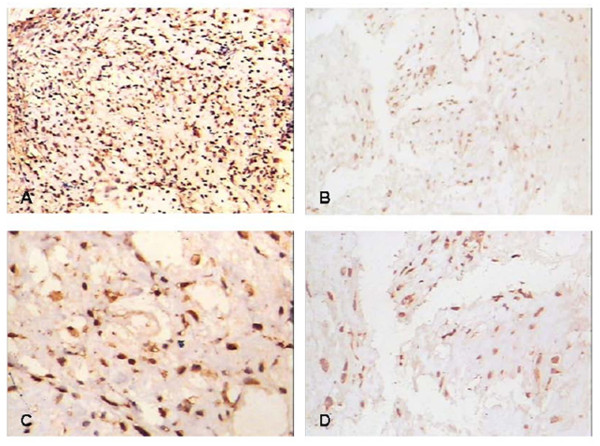
**The expression of IDH1 and p53 in low histological Rosen grade biopsy**. IDH1 expresses at high level accompanying with high expressed p53 in Low histological Rosen grade biopsy.(A) Expression of IDH1 in low histological Rosen grade biopsy, × 100;(B) Expression of p53 in low histological Rosen grade biopsy, × 100; (C) Expression of IDH1 in low histological Rosen grade biopsy, × 200;(D) Expression of p53 in low histological Rosen grade biopsy, × 200.

**Figure 5:**
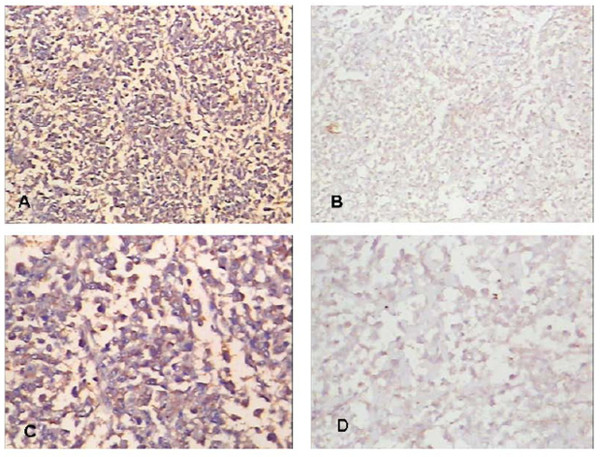
**The expression of IDH1 and p53 in high histological Rosen grade biopsy**. IDH1 expresses at low level accompanying with low expressed p53 in high histological Rosen grade biopsy.(A) Expression of IDH1 in high histological Rosen grade biopsy, × 100;(B) Expression of p53 in high histological Rosen grade biopsy, × 100; (C) Expression of IDH1 in high histological Rosen grade biopsy, × 200;(D) Expression of p53 in high histological Rosen grade biopsy, × 200.

**Figure 6:**
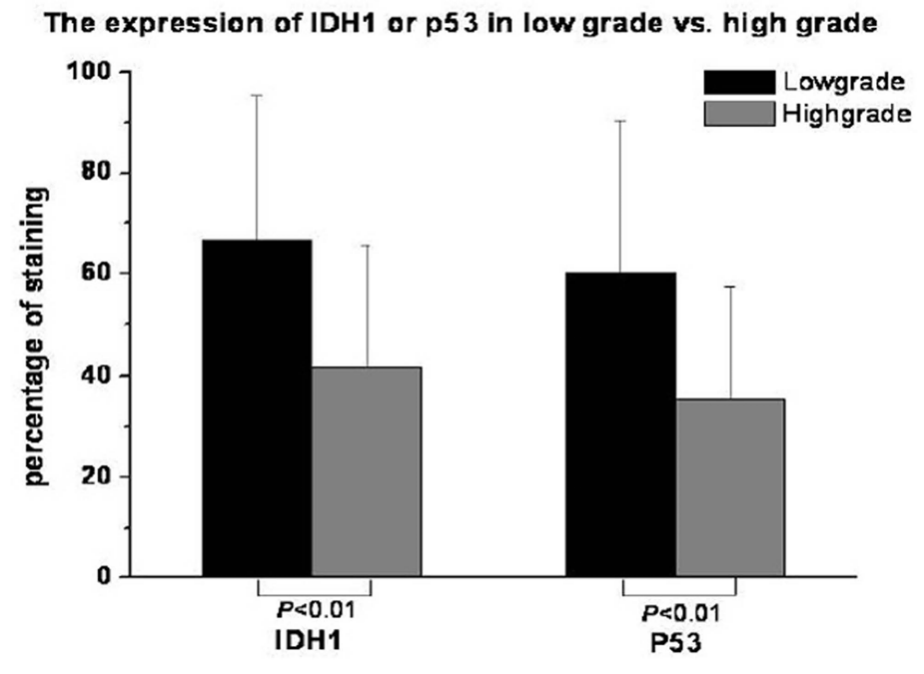
**The immunostaining percentages of IDH1 and p53 in low Rosen grade vs. high Rosen grade**. IDH1 expresses higher in Low histological Rosen grade compare with high histological Rosen grade at the level of the immunostaining percentages (*P *< 0.01), so does p53 (*P *< 0.01).

**Figure 7:**
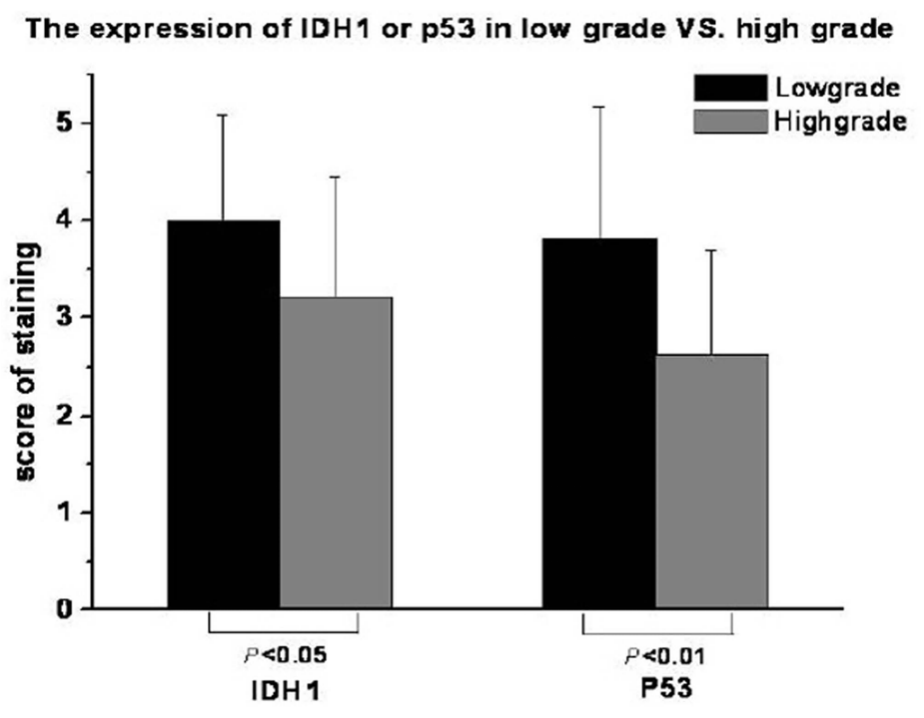
**The immunostaining scores of IDH1 and p53 in low Rosen grade vs. high Rosen grade**. IDH1 expresses higher in Low histological Rosen grade compare with high histological Rosen grade at the level of the immunostaining scores (*P *< 0.05), so does p53 (*P *< 0.01).

**Figure 8:**
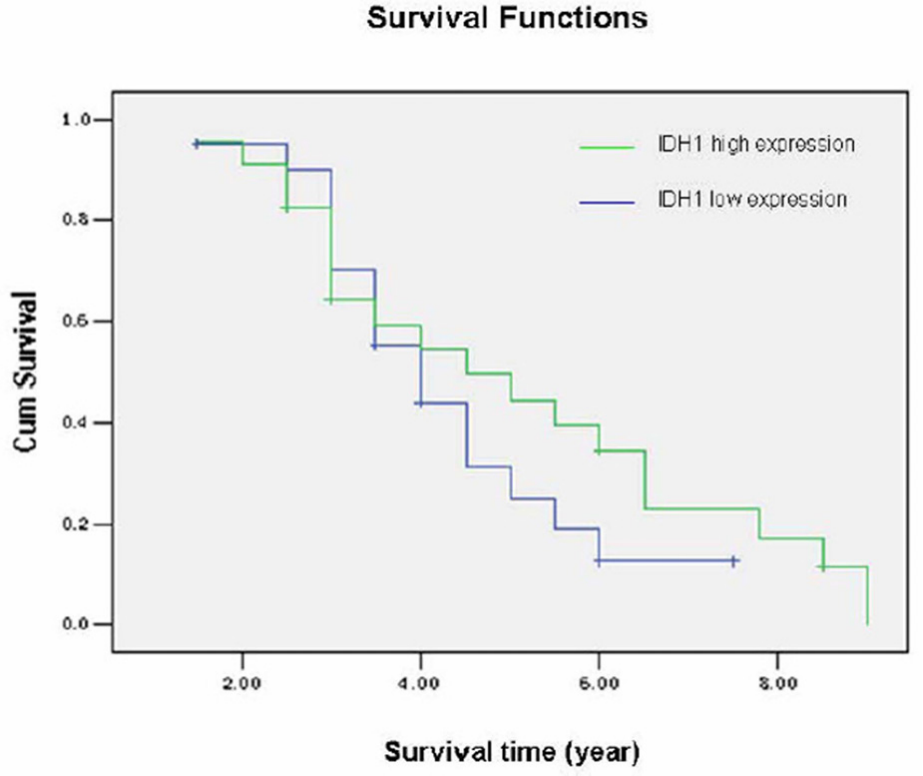
**The relationship between IDH1 and survival**. The IDH1 high expression group represents the osteosarcoma patients with >50% IDH1 positive staining. Patients with ≤ 50% IDH1 positive staining are recorded as low-expression group. The survival time in the *χ *-axis was given as years. There is no significant correlation between IDH1 expression and overall survival (*P *= 0.342).

IDH1 mainly locates on the cytoplasm (Such as Fig. 1A, Fig. 4A, and Fig. 5A). It's positive expression was identified using immunohistochemistry in 40 of 44 (90.9%) osteosarcoma tumors, of which 23 of 44 (52.2%) exhibits high staining (Table 2). The average IDH1 immunostaining percentage is 53.57%(SD: 28.99%, range from 8% to 100%). The average score is 3.59 (SD: 1.22, range from 1 to 5). IDH1 expresses higher in low Rosen grade osteosarcoma vs. high Rosen grade osteosarcoma [30-32] (Fig. 4, Fig. 5, Fig. 6, and Fig. 7). IDH1 correlates with metastasis negatively (*P *= 0.016, r = -0.361). There is no significant correlation between IDH1 expression and overall survival (*P *= 0.342) (Fig. 8).

**Table 2:** The expression of IDH1 and P53 in osteosarcoma biopsies

Proteins*	Expression**							Positive N***
		
	1	2	3	4	5	Low	High	
	N (%)	N (%)	N (%)	N (%)	N (%)	N (%)	N (%)	N (%)
IDH1	4 (9.1)	2 (4.5)	15 (34.1)	10 (22.7)	13 (29.5)	21 (47.7)	23 (52.2)	40 (90.9)
P53	7 (15.9)	6 (13.6)	12 (27.3)	10 (22.7)	9 (20.5)	25 (56.8)	19 (43.2)	37 (84.1)

* P < 0.01(p = 0.000) r = 0.620, IDH1 correlates with P53 positively; Spearman's rho.

** P > 3/40.05(P = 0.316), IDH1 vs. P53; Mann-Whitney U.

*** P > 3/40.05(0.334), IDH1 vs. P53; Pearson Chis-square test;

### P53 correlates with histological Rosen grade, metastasis and overall survival in clinical osteosarcoma biopsies

**Figure 9:**
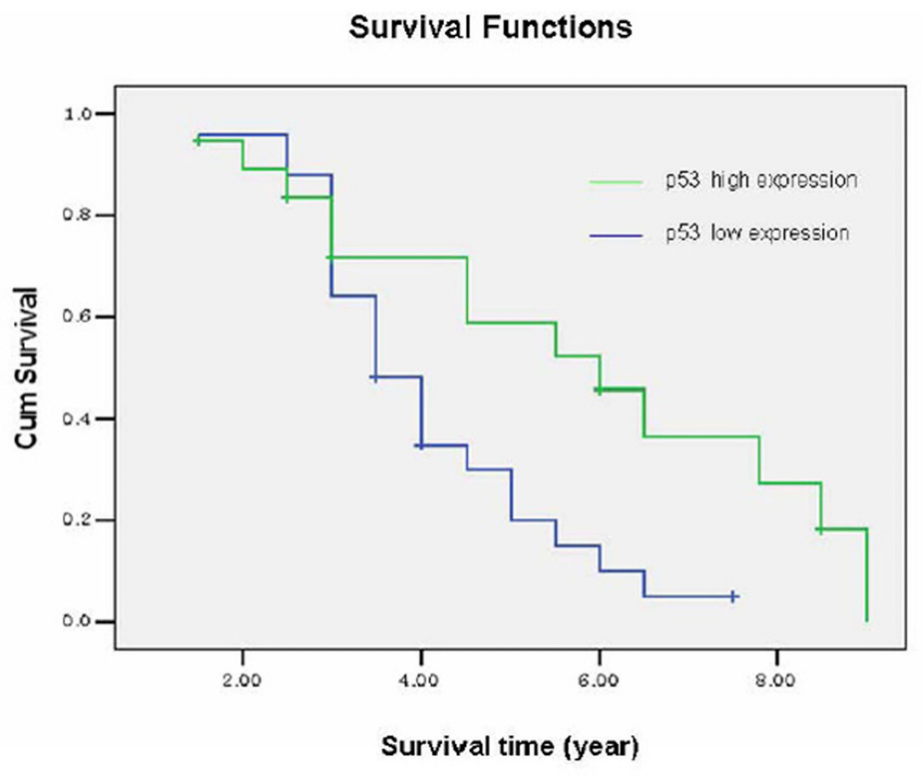
**The relationship between p53 and survival**. The p53 high expression group represents the osteosarcoma patients with >50% p53 positive staining. Patients with ≤ 50% p53 positive staining are recorded as low-expression group. The survival time in the *χ*-axis was given as years. High-expression p53 patients have better survival than low-expression p53 patients do (*P *= 0.019).

P53 mainly locates on the nuclear (Such as Fig 4B, Fig 4D), Its positive expression is identified using immunohistochemistry in 37 of 44 (84.1%) osteosarcoma tumors, of which 19 of 44 (43.2%) exhibits high staining (Table 2). The average p53 immunostaining percentage is 47.25%(SD: 28.82%, range from 4.5% to 100%). The average score is 3.18 (SD: 1.35, range from 1 to 5). P53 expresses higher in low Rosen grade osteosarcoma (Fig. 4, Fig. 5, Fig. 6, Fig. 7). P53 correlates with metastasis negatively (P = 0.001, r = -0.473). High-expression p53 patients have better survival than low-expression p53 patients do (P = 0.019) (Fig. 9).

### IDH1 correlates with p53 in clinical osteosarcoma biopsies

There is no significant difference between IDH1 and p53 in clinical osteosarcoma biopsies. Positive correlation between IDH1 and p53 expression is demonstrated in our study (Table 2, Fig. 4, and Fig. 5).

## Discussion

IDH1 catalyzes decarboxylation of isocitrate into alpha-ketoglutarate 16. Shechter et al. [17] described that activity of IDH1 is coordinately regulated with the cholesterol and fatty acid biosynthetic pathways, suggesting that IDH1 provides NADPH required by these pathways. It was described IDH1 appears to function as a tumor suppressor that, when mutationally inactivated, contributes to tumorigenesis [22]. IDH1 is likely to function as a tumor suppressor gene rather than as an oncogene [22]. IDH1, encoding two TCA enzymes, fumarate hydratase (FH) and succinate dehydrogenase (SDH), has been found to sustain loss-of-function mutations in certain human tumors, which likewise contribute to tumor growth via stimulating the HIF-1a pathway and mutationally altering metabolic enzymes [33,34]. As IDH1 also catalyzes the production of NADPH, it is possible that a decrease in NADPH levels resulting from IDH1 mutation contributes to tumorigenesis through effects on cell metabolism and growth [17]. Zhao et al. [22] showed that mutation of IDH1 impairs the enzyme's affinity for its substrate and dominantly inhibits wild type IDH1 activity with the formation of catalytically inactive heterodimers. Mutation of the IDH1 gene was strongly correlated with a normal cytogenetic status [21].

In this study, we firstly demonstrate that IDH1 is detected in U2OS with wild type p53 and MG63 with mutation p53 by immnohistochemistry, Realtime-PCR and Western Blotting. Intriguingly, our study demonstrates that IDH1 markedly increases in U2OS compare with MG63 not only in mRNA level but also in protein level. It is conceivable that the expression of IDH1 may relate to p53.

Human osteosarcoma cell line MG63 was found with Deletion and rearrangement of the p53 gene [35-37]. No Wild type p53 expression could be detected in this cell line. Our results are in accordance with the results of Masuda et al. [6] and Mulligan et al. [36] and indicate that inactivation of p53 is a common event in osteosarcoma development. In addition, we authenticate the wild type p53 in human osteosarcoma cell line U2OS in our study.

P53 is described as a tumor suppressor in many tumors. Culotta and Koshland [38] and Harris et al [39] gave an extensive account of its discovery and function as well as the use of p53 in cancer risk assessment. Activity of p53 ubiquitously lost in osteosarcoma either by mutation of the p53 gene itself or by loss of cell signaling upstream or downstream of p53 [40]. Xue et al. [41] reported that p53 inactive may be required for maintenance of aggressive tumors. Marion et al. [42] showed that p53 is critical in preventing the generation of human pluripotent cells from suboptimal parental cells. Harris and Hollstein [39] highlighted the clinical implications of changes in the p53 gene in the pathogenesis, diagnosis, prognosis, and therapy of human cancer. But, little is known about the combinatory role of p53 and IDH1 in OS cells. We are curious about the role of p53 and IDH1 in osteosarcoma.

Most studies addressing the immunohistochemical expression of IDH1 or p53 in biopsies have used a semiquantitative scoring approach of the staining results [43-47], often with a 10% threshold for scoring a tumor as positive and with a 50% threshold for scoring a tumor as high expression level [48]. Using this approach, the immunoreactivity for IDH1 or p53 has been used to investigate its correlation with clinical features [47]. The staining pattern, and thus the difference in IDH1 reactivity, is highly different among individual tumors, showing a range from 8% through 100% IDH1-positive tumor cells, while the P53, ranging from 5% to 100%. In addition, the positive rate of IDH1 is 90.9%, while the p53 is 84.1%. The high staining rate of IDH1 is 52.2%, while the p53 is 43.2%. Furthermore, IDH1 expresses higher in patients with low histological Rosen grade. IDH1 correlates with metastasis negatively. There is no significant correlation between IDH1 expression and overall survival. In our study, lower IDH1 expression in higher Rosen grade may not convey mutation in the gene. To substitute, genetic studies of IDH1 gene alteration may be of value. The study is limited by the fact that there were only 44 patients and without intimate following up information. However, it may, from the theoretical point of view, still be valuable to study the role of IDH1 in osteosarcoma. In accordance with former results, p53 in our osteosarcoma patients correlates with histological Rosen grade, metastasis and overall survival. In our study, the expression of IDH1 does not correlate some other clinical features such as age, localization of primary tumor and histological type.

Interestingly, patients in our study with High expression of IDH1 had a very high p53 expression in osteosarcoma biopsies, which is accordance with our result in osteosarcoma cell lines MG63 and U2OS. A recent study has shown IDH1 appears to function as a tumor suppressor contributes to tumorigenesis in part through induction of the HIF-1 pathway [22]. Parsons et al. [20] found that IDH1 mutations had a very high frequency of p53 mutation in human glioblastoma. Accumulation of functional p53 protein followed by p53-dependent apoptosis has been described in cultured cells exposed to hypoxia [49]. P53 inhibits HIF-1 dependent transcription and decrease the chances of normal cells surviving under hypoxia since the expression of most glycolytic enzymes is HIF-1 dependent [50]. It is conceivable that IDH1 may relate to p53 with the function of HIF-1.

## Conclusions

IDH1 may correlate with p53 and be a biomarker for osteosarcoma correlate with histological Rosen grade and metastasis.

## List of abbreviations

IDH1: isocitrate dehydrogenase 1; p53: transformation-related protein 53; OS: osteosarcoma; PTEN: phosphates and tensin homolog; Rb: retinoblastoma gene; TCA: The citric acid cycle; SD: Std. Deviation.

## Competing interests

The authors declare that they have no competing interests.

## Authors' contributions

Hu X carried out most parts of the experiment; Qi BW, Fu T, Wu G, Zhou M, Luo J and Xu JH participated in the experiment; Yu AX conceives the study project, organizes the whole study process, provides financial support, and finalizes the manuscript. All authors have read and approved the final manuscript.
